# Robust, Thermo-Malleable, and Closed-Loop Recyclable Mulberry Paper/Polyimine Composite Films Enabled by Dynamic Covalent Interpenetrating Networks

**DOI:** 10.3390/ma19112310

**Published:** 2026-05-29

**Authors:** Yisheng Liao, Yongguang Huang, Peipei Cheng, Hao Huang, Ling Liang, Lin Fan, Hongfang Lai, Guocui Qi, Dexiu Min, Xiaodong Li, Chengyu Wang, Feng Liu

**Affiliations:** 1 Guangxi Key Laboratory of Sericulture Ecology and Intelligent Technology Application, Guangxi Collaborative Innovation Center of Modern Sericulture and Silk, Guangxi Colleges Universities Key Laboratory of Exploitation and Utilization of Microbial and Botanical Resources, School of Chemistry and Bioengineering, Hechi University, Hechi 546300, China; 2 School of Teacher Education, Hechi University, Hechi 546300, China; 3 Shandong Xingang Co., Ltd., Linyi 276000, China; 4 Key Laboratory of Bio-Based Material Science and Technology of Ministry of Education, Northeast Forestry University, Harbin 150040, China; wangcy@nefu.edu.cn

**Keywords:** mulberry paper, polyimine vitrimer, composite films, dynamic covalent networks, thermo-malleability, closed-loop recycling

## Abstract

The persistence of petrochemical plastics necessitates high-performance and recyclable alternatives, yet balancing mechanical robustness with component-level closed-loop recovery remains challenging for biomass-based plastic-replacement films. Here, a high-performance, thermo-malleable, and closed-loop recyclable composite film is constructed by integrating a highly crystalline enzyme-treated mulberry paper (Enzyme-MP) fiber network with an in situ formed polyimine (PI) vitrimer network via capillary-assisted infiltration. This process induces densification and extensive interfacial hydrogen bonding, forming a confined interpenetrating architecture that enhances stress transfer and restricts chain mobility. As a result, the composite film achieves a tensile strength of 70.3 MPa and a Young’s modulus of 2.37 GPa, together with excellent thermomechanical stability over a broad temperature range. The dynamic imine exchange enables thermo-malleability, allowing seamless self-welding and thickness-scalable lamination at 120 °C. The dense structure also acts as an effective barrier, reducing water uptake to 14.3% and providing resistance to various organic solvents. Furthermore, full-component closed-loop recycling is realized via room-temperature transimination, enabling selective depolymerization of the matrix while preserving the crystalline cellulose fiber network. This work demonstrates a viable strategy to integrate high-strength film performance, processability, and chemical recyclability in biomass-based composite films, while providing a basis for future cradle-to-cradle material circulation in recyclable plastic-replacement films.

## 1. Introduction

The extensive consumption and improper disposal of conventional petrochemical plastics have led to severe environmental challenges, thereby necessitating the development of sustainable alternatives. Biomass-derived materials, as the most abundant renewable and biodegradable resources in nature, have shown great potential to replace petroleum-based plastics in next-generation high-performance film and sheet applications [1,2]. However, the fabrication of high-performance biomass composite films critically depends on the selection of robust fiber networks and the effective integration of multiphase interfaces.

Mulberry bark paper, a representative product of traditional Chinese papermaking, exhibits excellent folding endurance, tensile strength, and long-term stability, as evidenced by its application in the preservation of ancient manuscripts [3]. From a materials perspective, such durability originates from the intrinsic characteristics of mulberry fibers, including their high aspect ratio, high crystallinity, and strongly interwoven three-dimensional network [4]. These features make mulberry fibers promising fibrous building blocks for advanced plastic-replacement films [5,6,7]. In particular, enzyme-treated mulberry paper (Enzyme-MP), prepared via mild biological degumming, retains a highly robust fibrous network together with abundant surface hydroxyl groups that are favorable for interfacial interactions [8]. Nevertheless, pristine natural mulberry paper relies primarily on a hydrogen-bonding network, which results in limited dimensional stability and poor resistance to fluid attack under humid or chemically complex environments [9]. Conventional modification strategies typically involve the impregnation of natural fiber materials with thermosetting polymers, such as polyurethane, to enhance tensile integrity and environmental stability [10,11,12]. Although effective in improving strength and barrier-related performance, the formation of permanent covalent crosslinked networks inevitably sacrifices processability and recyclability [13,14]. Consequently, end-of-life composite films are often subjected to landfill or incineration, leading to both resource waste and environmental burden, which fundamentally contradicts the principles of a circular economy [15].

Vitrimers enabled by dynamic covalent chemistry offer a promising route to reconcile performance and recyclability [16,17]. In particular, polyimine (PI) systems based on dynamic Schiff-base chemistry can undergo bond exchange or depolymerization under thermal or chemical stimuli, enabling topological rearrangement, reprocessing, and chemical regeneration [18,19,20]. However, translating vitrimer chemistry into multiphase biomass film materials remains challenging. A dense polymer–fiber architecture is required to provide tensile reinforcement, barrier performance, and dimensional stability, while the dynamic network must remain accessible for mild and efficient closed-loop recycling [21,22,23].

Herein, we propose a dynamic interfacial engineering strategy to construct a high-strength, thermo-malleable, and chemically recyclable biomass-based composite film. Enzyme-MP is employed as a continuous cellulose fiber network, and a low-viscosity PI precursor is introduced into the porous fiber network through a hot-press-assisted infiltration. Capillary densification and extensive interfacial hydrogen bonding between cellulose hydroxyl groups and imine-containing polymer chains generate a dense fiber-vitrimer interpenetrating architecture [24]. This confined structure significantly enhances stress transfer and restricts polymer chain mobility, while preserving the dynamic reversibility of imine chemistry within the composite film. The optimized Enzyme-MP/PI composite film achieves a tensile strength of 70.3 MPa, corresponding to an approximately 195% increase relative to neat PI, together with stable thermomechanical behavior and strong solvent resistance. Benefiting from dynamic imine exchange, the material demonstrates thermo-malleability, allowing seamless lamination at 120 °C for thickness customization. In addition, a mild room-temperature aminolysis strategy enables nondestructive separation of the highly crystalline cellulose fiber network and chemical regeneration of the polymer matrix, thereby demonstrating component-level closed-loop recovery with minimal loss of tensile performance after multiple cycles. This work provides a practical strategy for designing high-performance, reprocessable, and recyclable biomass-based composite films as potential plastic-replacement materials.

## 2. Materials and Methods

### 2.1. Materials

Mulberry branches were collected from a local sericulture plantation in Yizhou, Guangxi, China. Pectinase, laccase, sodium hydroxide (NaOH), sodium sulfite (Na_2_SO_3_), sodium carbonate (Na_2_CO_3_), disodium ethylenediaminetetraacetate (EDTA-2Na), fatty alcohol polyoxyethylene ether (AEO), sodium dodecylbenzene sulfonate, terephthalaldehyde (TPTA), 1,5-pentanediamine, and tris(2-aminoethyl)amine (TREN) were purchased from Shanghai Aladdin Biochemical Technology Co., Ltd. (Shanghai, China). All reagents were used as received without further purification.

### 2.2. Preparation of Degummed Mulberry Fibers and Paper Sheets

Mulberry branches were first soaked in deionized water for 72 h to facilitate peeling of the bark and removal of the green outer epidermis, affording the bast layer of the mulberry bark. The obtained bark was then dried at room temperature for 24 h.

**Figure 1:**
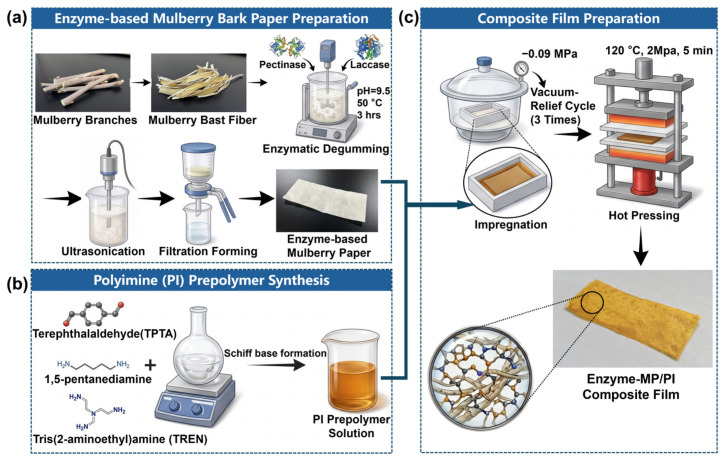
Fabrication strategy and interpenetrating network formation of the Enzyme-MP/PI composite films. (**a**) Enzymatic degumming process for producing enzyme-treated mulberry paper (Enzyme-MP) with a preserved highly crystalline fiber network. (**b**) Synthesis of the polyimine (PI) vitrimer prepolymer via Schiff-base condensation. (**c**) Vacuum-assisted impregnation and hot-pressing process, illustrating capillary-driven infiltration and the formation of a densified fiber-vitrimer interpenetrating architecture.

For enzymatic degumming, the dried bast fibers were pretreated in an aqueous EDTA-2Na solution (6 g/L) at 100 °C for 2 h. After cooling to 50 °C, the pH was adjusted to 9.5, followed by the addition of pectinase (40 g/L) and laccase (2 g/L), and the mixture was maintained for 3 h (Figure 1a). The fibers were then cooked in a mixed solution containing penetrant AEO (2 g/L), sodium dodecylbenzene sulfonate (3 g/L), Na_2_SO_3_ (2 g/L), Na_2_CO_3_ (3 g/L), and NaOH (5 g/L) at 100 °C for 3 h.

For alkaline degumming, used as a control, the mulberry bast fibers were first cooked in a 15 g/L NaOH solution at 100 °C for 6 h to remove most non-cellulosic components and promote initial fiber separation, followed by thorough washing to neutrality to remove dissolved impurities and residual alkali. A second, shorter cooking step was then conducted in NaOH solution of the same concentration at 100 °C for 1.5 h to further complete degumming under relatively mild stepwise conditions. The fibers were washed with water to neutrality after the final treatment.

The enzyme-treated and alkali-treated fibers were then mechanically beaten for 40 min, ultrasonically dispersed in water for 5 min at a solid-to-liquid ratio of 1:70, and collected by filtration. The resulting wet sheets were dried at 30 °C for 12 h to obtain enzyme-treated mulberry paper sheets (Enzyme-MP) and alkali-treated mulberry paper sheets (Alkali-MP), respectively.

### 2.3. Synthesis of the Polyimine (PI) Prepolymer

The PI prepolymer was synthesized via a catalyst-free Schiff-base condensation reaction at room temperature. TPTA (1.00 g, 7.45 mmol) was dissolved in a mixed solvent of ethanol (7.5 mL) and dichloromethane (DCM, 12.5 mL). Subsequently, 1,5-pentanediamine (0.228 g, 2.23 mmol) and TREN (0.509 g, 3.48 mmol) were added sequentially at a molar ratio of 3:0.9:1.4. The mixture was magnetically stirred for 5 min to obtain the PI prepolymer solution (Figure 1b).

For preparation of neat PI films, the resulting solution was cast into a polytetrafluoroethylene mold and allowed to evaporate in a fume hood for 12 h. The film was then sequentially cured at 78 °C for 3 h, 95 °C for 1 h, and 105 °C for 1 h.

### 2.4. Preparation of the Enzyme-MP/PI Composite Films

The Enzyme-MP/PI composite films were fabricated by a vacuum-assisted impregnation and hot-pressing process (Figure 1c). Dried Enzyme-MP sheets were placed in a polytetrafluoroethylene mold and immersed in the PI prepolymer solution. To ensure sufficient precursor penetration into the porous fiber network, three vacuum–release cycles were conducted, during which the pressure was reduced by 0.09 MPa relative to atmospheric pressure. The impregnated Enzyme-MP sheets were then dried at room temperature for 12 h to remove the solvent, yielding precured composite sheets. By varying the number of impregnation cycles from 2 to 10, the Enzyme-MP/PI mass ratio was adjusted from 1:0.5 to 1:4.5.

The precured samples were subsequently sandwiched between polytetrafluoroethylene films and hot-pressed at 120 °C under 2 MPa for 5 min to achieve in situ crosslinking and film densification. The samples were demolded after cooling to below 80 °C.

### 2.5. Characterization

The morphology of the samples was observed by SEM (ZEISS GeminiSEM 300, Carl Zeiss AG, Oberkochen, Germany) at 20 kV after gold sputter-coating. FTIR spectra were recorded using a Nicolet iS50 spectrometer over 4000–500 cm^−1^ with a resolution of 4 cm^−1^ and 32 scans. XRD patterns were collected on a Rigaku MiniFlex600 diffractometer (Rigaku Corporation, Tokyo, Japan) using Cu Kα radiation (λ = 1.5406 Å) at 40 kV and 40 mA, over 2*θ* = 5–50° at 5° min^−1^. Uniaxial tensile tests were performed on an Instron 5565 universal testing machine (Instron Corporation, Norwood, MA, USA) at room temperature. Rectangular film specimens (150 mm × 10 mm, with measured thicknesses ranging from 0.12 mm to 0.22 mm) were stretched at a crosshead speed of 5 mm min^−1^ using a gauge length of 50 mm. The thickness of each specimen was measured individually before testing, and at least five specimens were tested for each sample. DMA was performed on a TA Q800 (TA Instruments, New Castle, DE, USA) using a tension film clamp in tensile mode. Film specimens (20 mm × 5 mm) were tested from 30 °C to 120 °C at 5 °C min^−1^ and 1 Hz, with a strain amplitude of 0.1% within the linear viscoelastic region; the measured thickness of each specimen was input before testing. At least two measurements were conducted for each formulation. TGA was performed on a TA Instruments 550 analyzer (TA Instruments, New Castle, DE, USA) under nitrogen flow. Samples (~4 mg) were heated from 25 °C to 800 °C at 10 °C min^−1^ under a nitrogen flow rate of 50 mL min^−1^.

## 3. Results and Discussion

### 3.1. Selective Deconstruction and Structural Preservation of Mulberry Fiber Networks

To elucidate the deconstruction mechanisms induced by different degumming strategies, the morphology, molecular composition, and crystalline structure of mulberry bark paper prepared via alkaline (Alkali-MP) and enzymatic (Enzyme-MP) treatments were systematically investigated.

**Figure 2:**
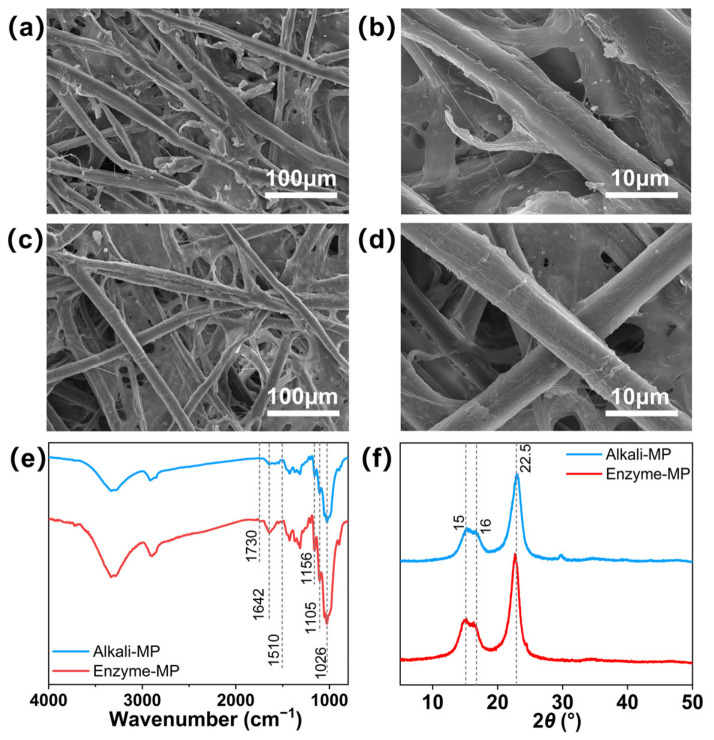
Comparative morphological, chemical, and crystalline characterization of Alkali-MP and Enzyme-MP. (**a**,**b**) Low- and high-magnification SEM images of Alkali-MP. (**c**,**d**) Low- and high-magnification SEM images of Enzyme-MP, showing preserved fiber integrity and an interwoven porous network. (**e**) FTIR spectra demonstrating effective removal of non-cellulosic components. (**f**) XRD patterns confirming the retention of the cellulose I crystalline structure.

SEM observations (Figure 2) reveal that non-cellulosic binding components were effectively removed from the fiber surfaces in both cases, resulting in smooth, cylindrical fibers with high aspect ratios that interweave into a continuous three-dimensional porous network. Notably, no apparent microcracks or excessive fibrillation damage were observed under controlled alkaline and enzymatic treatment (Figure 2b,d). This comparison indicates that the mild enzymatic process achieves degumming efficiency comparable to alkaline treatment, which is attributed to its selective degradation of amorphous matrix components while preserving the morphological integrity of the fiber scaffold [25,26].

FTIR spectra (Figure 2e) further confirm the effective removal of non-cellulosic constituents. The complete disappearance of characteristic peaks at 1730–1740 cm^−1^ (C=O stretching of pectin/hemicellulose) and 1510 cm^−1^ (aromatic skeletal vibration of lignin) indicates efficient elimination of these components [27,28]. Meanwhile, the retention of cellulose-specific peaks at 1156 and 1105 cm^−1^ (β-1,4-glycosidic bonds) and 1026 cm^−1^ (C–O/C–C stretching) demonstrates that the cellulose backbone remains intact during the degumming process [29]. The absorption band at 1642 cm^−1^ is attributed to physically adsorbed water [30].

XRD analysis (Figure 2f) provides further insight into the supramolecular organization of the degummed mulberry fibers. The diffraction features were indexed by comparison with literature-reported cellulose I patterns, and the interplanar spacings were calculated using Bragg’s equation, d = λ/(2sin *θ*), where λ = 1.5406 Å for Cu Kα radiation [31]. Both Alkali-MP and Enzyme-MP retain the characteristic diffraction signals of native cellulose I, including a sharp main peak at 2*θ* ≈ 22.5° and a broad, partially overlapped diffraction band at 15–16°. These reflections are commonly indexed as the (200) plane and the overlapped (1$\bar{1}$0)/(110) reflections of cellulose I, respectively, with corresponding d-spacings of approximately 3.95 Å and 5.4–5.9 Å. Native cellulose from higher plants is generally considered to be rich in the cellulose Iβ allomorph, which has been reported to adopt a monoclinic crystal structure with the P2_1_ space group [32]. However, because the present laboratory XRD patterns contain broad and partially overlapped reflections typical of semicrystalline cellulose, the peak assignments are used here to confirm the retention of cellulose I-type crystallinity rather than to perform full crystal-structure refinement. The calculated crystallinity indices (CrI) reach 87.3% and 87.5% for Alkali-MP and Enzyme-MP, respectively. This high crystallinity indicates the relative enrichment of ordered crystalline domains after the removal of amorphous non-cellulosic components [33,34]. Importantly, no diffraction peak splitting or shift associated with cellulose II formation is observed, suggesting that the degumming conditions effectively avoid mercerization-induced irreversible lattice transformation [35,36]. Overall, in contrast to the conventional alkaline degumming strategy, the enzymatic degumming strategy enables the selective removal of amorphous constituents while maintaining chemical inertness toward the highly crystalline cellulose domains. The resulting highly crystalline, morphology-preserved, and continuously interconnected fiber network provides an ideal reinforcing scaffold for constructing high-performance polyimine vitrimer composite films.

### 3.2. Resin Content–Dependent Formation of Densified Interpenetrating Composite Films

**Figure 3:**
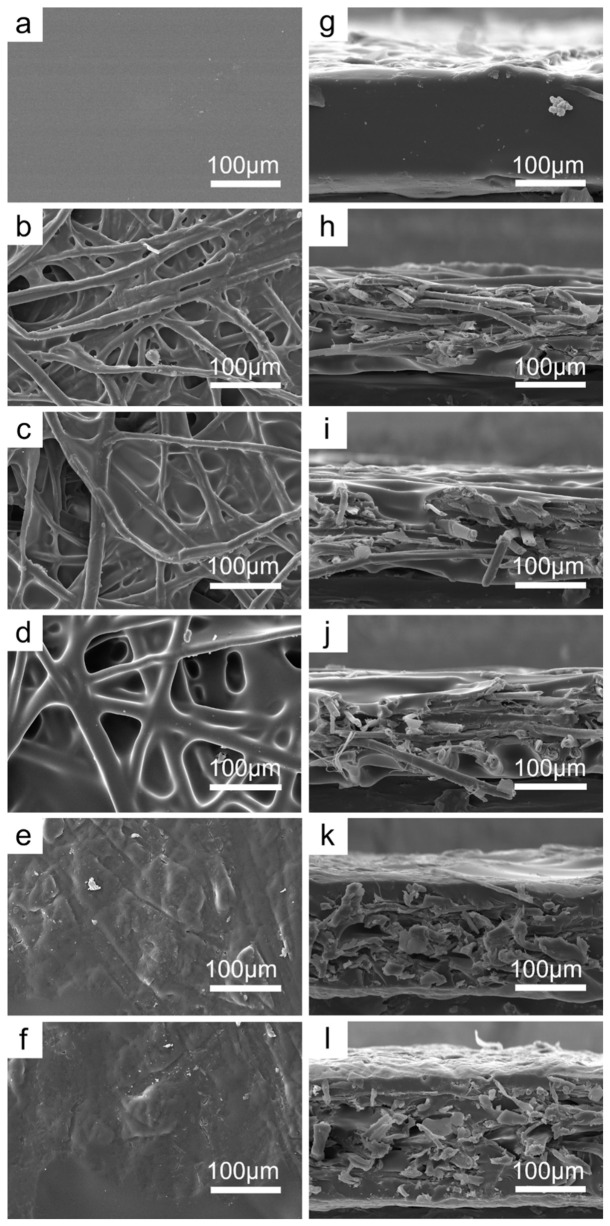
Morphological evolution and interpenetrating architecture of Enzyme-MP/PI composite films with varying mass ratios. Surface and tensile-fractured cross-sectional SEM images of (**a**,**g**) neat PI, and composite films with Enzyme-MP/PI mass ratios of (**b**,**h**) 1:0.5, (**c**,**i**) 1:1.5, (**d**,**j**) 1:2.5, (**e**,**k**) 1:3.5, and (**f**,**l**) 1:4.5. The images illustrate progressive capillary densification, interface formation, and the development of a densified fiber-vitrimer interpenetrating morphology.

A series of Enzyme-MP/PI composite films with paper-to-resin mass ratios ranging from 1:0.5 to 1:4.5 were prepared by varying the number of PI prepolymer impregnation cycles from 2 to 10. SEM observations (Figure 3) reveal that resin infiltration plays a decisive role in regulating the interfacial morphology and film-level densification of the composite system.

The neat PI film as a whole exhibits a typical dense and homogeneous morphology. At low resin loadings (1:0.5 and 1:1.5), the woven contours of the fibrous network remain visible on the composite surface (Figure 3b,c), while micropores in the fracture cross-sections indicate that a continuous resin matrix has not yet been fully established (Figure 3h,i). When the mass ratio is increased to 1:3.5, capillary-driven infiltration under hot pressing allows the liquid PI precursor to thoroughly permeate the porous fiber network, resulting in a dense and smooth surface morphology (Figure 3e). No obvious fiber pull-out or phase-separated gaps are observed on the fracture cross-section (Figure 3k), confirming excellent interfacial compatibility between the two phases. This behavior is attributed to the strong intermolecular interactions between polar imine-containing polymer chains and abundant hydroxyl groups on the cellulose surface, which promote the formation of a robust fiber-vitrimer interpenetrating architecture. In contrast, excessive resin loading (1:4.5) produces a thick PI overlayer on the surface (Figure 3f) and a pronounced resin-rich region in the cross-section (Figure 3l), indicating heterogeneous phase accumulation that may weaken the tensile reinforcement efficiency of the rigid fiber network.

**Figure 4:**
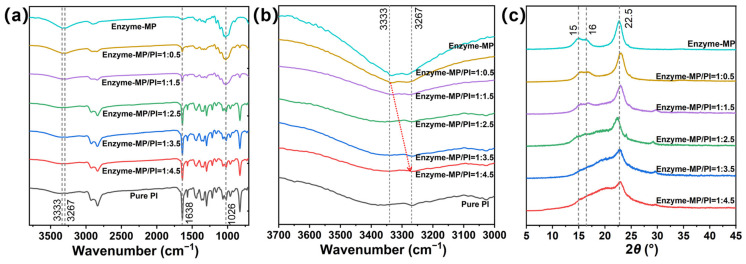
Spectroscopic and crystallographic evidence of interfacial interactions in Enzyme-MP/PI composite films. (**a**) FTIR spectra of neat PI, Enzyme-MP, and composite films with varying mass ratios. (**b**) Enlarged FTIR spectra (3100–3600 cm^−1^) highlighting the red-shift of –OH stretching vibrations induced by interfacial hydrogen bonding. (**c**) XRD patterns demonstrating the preservation of the cellulose crystalline features and the evolution of apparent crystallinity.

FTIR spectra (Figure 4a,b) further verify the noncovalent interfacial interaction mechanism. Pristine Enzyme-MP exhibits a broad cellulose –OH stretching band centered at 3333 cm^−1^, whereas neat PI shows the characteristic absorption of dynamic imine bonds (–C=N–) at 1638 cm^−1^ [37,38]. In the composite spectra, the characteristic imine peak of PI coexists with the cellulose backbone vibrations, such as the C–O–C stretching at 1026 cm^−1^, indicating that the hot-press curing process preserves the natural rigid fiber network of Enzyme-MP. More importantly, as the PI content increases, the –OH stretching band progressively broadens and its center shifts to lower wavenumbers, accompanied by strong coupling with the low-frequency broad band of PI at 3267 cm^−1^ (Figure 4b) [22]. This redshift provides direct spectroscopic evidence for strengthened interfacial hydrogen bonding [21,39]. Specifically, the free hydroxyl groups enriched on the cellulose surface act as proton donors, whereas the electronegative imine groups and adjacent nitrogen-containing bonds in the PI network act as proton acceptors, thereby establishing a dense hydrogen-bonding network across the interface. Such interfacial hydrogen bonds function as dynamic molecular anchors within the interpenetrating architecture. This interpretation is consistent with the seamless fracture morphology observed by SEM (Figure 3k) and further suggests that the interface can dissipate fracture energy through repeated bond rupture and reformation under mechanical loading. Therefore, the optimized resin content not only enables full infiltration of the porous Enzyme-MP network but also maximizes interfacial hydrogen bonding, thereby providing the molecular basis for efficient stress transfer and subsequent tensile reinforcement of the composite film.

XRD analysis (Figure 4c) further elucidates the evolution of supramolecular organization in the Enzyme-MP/PI composite films. The diffraction peaks were indexed according to the cellulose I assignments described above, with d-spacings calculated from Bragg’s equation using Cu Kα radiation. All samples, regardless of resin content, retain the characteristic cellulose I-type diffraction signals at 2*θ* ≈ 15–16° and 22.5°, corresponding to the overlapped (1$\bar{1}$0)/(110) reflections and the (200) reflection, respectively [31,32]. The corresponding d-spacings remain within the typical ranges of cellulose I, indicating that the in situ crosslinking of PI is sufficiently mild and does not disrupt the highly ordered crystalline domains of cellulose. Given the broad and overlapped nature of the cellulose reflections, these assignments are used to verify the preservation of cellulose I-type crystalline domains in the composite films rather than to claim complete crystallographic refinement. However, the apparent crystallinity index (CrI) decreases progressively with increasing PI content, from 87.5% for pristine Enzyme-MP to 63.0% at the highest resin loading. Here, CrI is used only as a semi-quantitative indicator of the relative diffraction contribution from cellulose crystalline domains in the multiphase composite films, rather than as an absolute crystallographic parameter. This reduction does not originate from crystal destruction, but rather from a phase dilution effect induced by incorporation of the amorphous PI matrix [21]. As the fraction of the amorphous phase increases, the relative volume fraction of the highly crystalline fiber network is physically diluted, leading to an overall decrease in diffraction intensity [22]. The resulting semi-crystalline/amorphous architecture enables effective microstructural complementarity. In this configuration, the dense crystalline cellulose domains act as rigid tensile reinforcements, whereas the low-modulus amorphous PI matrix buffers local stress concentrations and facilitates stress redistribution. Such synergistic reinforcement explains the remarkable tensile strength and mechanical integrity observed in the composite films [40].

### 3.3. Tensile Structure–Property Correlation and Fracture Mechanisms of Composite Films

**Figure 5:**
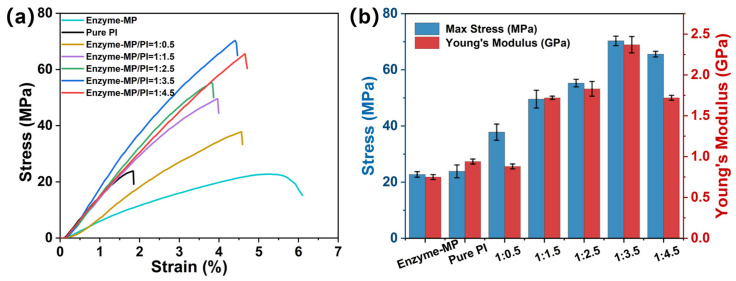
Tensile performance of Enzyme-MP/PI composite films as a function of resin content. (**a**) Representative engineering stress–strain curves of neat PI, Enzyme-MP, and composite films with different mass ratios. (**b**) Maximum tensile strength and Young’s modulus of the corresponding samples.

Uniaxial tensile testing reveals that resin content plays a decisive role in governing the tensile performance of the Enzyme-MP/PI composite films. As shown in Figure 5, pristine Enzyme-MP exhibits a tensile strength of only 22.7 MPa and a Young’s modulus of 0.75 GPa, which is mainly attributed to its porous internal structure and the associated stress concentration. Neat PI, as a flexible amorphous polymer, shows comparable values of 23.8 MPa and 0.94 GPa, respectively. After incorporation of the PI matrix, the tensile properties of the Enzyme-MP/PI composite films display a typical rise-and-fall trend with increasing resin content. Specifically, the tensile strength steadily increases from 37.8 MPa at a paper-to-resin mass ratio of 1:0.5 and reaches a maximum of 70.3 MPa at 1:3.5, while the Young’s modulus simultaneously increases to 2.37 GPa, corresponding to improvements of approximately 195% and 152%, respectively, compared with neat PI.

Fracture cross-sectional morphologies (Figure 3) further elucidate the evolution of stress transfer pathways and failure mechanisms under tensile loading. At low resin loadings (1:0.5 and 1:1.5), extensive fiber pull-out is clearly observed on the fracture surfaces (Figure 3h,i). This indicates that the discontinuous PI matrix is yielded prior to the fibrous network, resulting in a typical asynchronous fracture mode that limits synergistic reinforcement between the two phases [41,42]. In contrast, at the optimal mass ratio of 1:3.5, the fracture surface exhibits a synchronous fracture feature of the matrix and fibers, with no obvious phase separation or interfacial debonding (Figure 3k). This transition is attributed to the complete filling of micropores and inter-fiber voids within the fiber network by the low-viscosity PI precursor, together with the formation of abundant noncovalent interfacial hydrogen bonds between imine-containing chains and free hydroxyl groups on the cellulose surface [21,39]. Under tensile deformation, this dense hydrogen-bonding network functions as sacrificial bonds that dissipate fracture energy through repeated bond rupture and reformation [39,43]. Meanwhile, the continuous PI matrix serves as a stress-distribution phase, uniformly distributing the external stress to the rigid fiber network and thereby maximizing interfacial stress transfer efficiency.

However, further increasing the resin loading to 1:4.5 causes the tensile strength and Young’s modulus to decrease to 65.6 MPa and 1.85 GPa, respectively. This mechanical deterioration directly corroborates the phase dilution effect discussed above. Specifically, excessive amorphous PI forms a relatively thick resin-rich layer around the fiber network (Figure 3l), reducing the apparent crystallinity of the system to 63.0%. Under such conditions, the initial tensile deformation is dominated by the low-modulus polymer-rich phase, which substantially weakens the reinforcing efficiency of the rigid cellulose network. Taken together, these microstructural and tensile results indicate that an appropriate Enzyme-MP/PI ratio captures the optimal stiffness–strength balance within the semi-crystalline/amorphous multiphase architecture, thereby endowing the biomass composite film with considerable potential as a high-strength sustainable plastic replacement.

### 3.4. Thermomechanical Behavior and Interfacial Confinement Effects in Composite Films

**Figure 6:**
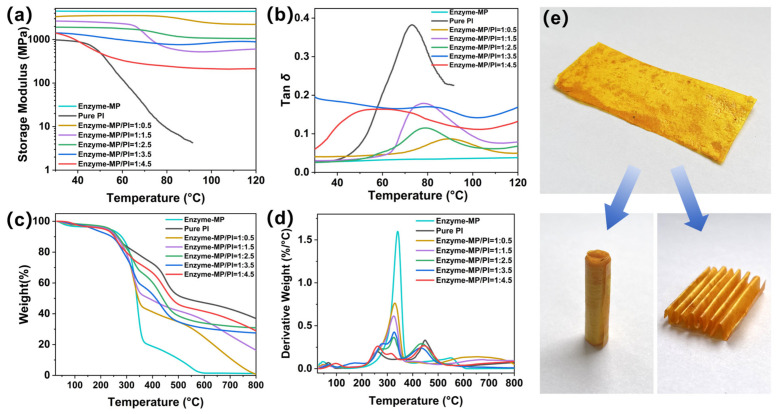
Thermomechanical behavior and thermal stability of Enzyme-MP/PI composite films. (**a**) Storage modulus and (**b**) loss tangent (tan δ) from DMA, showing interfacial confinement and restricted segmental relaxation. (**c**) TGA and (**d**) DTG curves indicating improved thermal stability. (**e**) Thermoforming demonstration of the Enzyme-MP/PI composite film with a mass ratio of 1:3.5 at 120 °C, showing reshaping into complex geometries.

Dynamic mechanical analysis (DMA) was employed to investigate the viscoelastic behavior and interfacial confinement mechanism of the materials over the key processing and service temperature range of 30–120 °C (Figure 6a,b). At 30 °C, within the glassy region, the neat polyimine vitrimer (PI) film exhibits a storage modulus of approximately 980 MPa, whereas the highly crystalline Enzyme-MP reaches 4482 MPa. With increasing PI content, the room-temperature storage modulus of the composite films gradually decreases from 3339 MPa at a paper-to-resin mass ratio of 1:0.5 to approximately 1400 MPa at 1:4.5. This trend can be attributed to the partial substitution of direct fiber–fiber hydrogen-bonding interactions with the softer amorphous PI phase following resin infiltration. The resulting phase-dilution effect reduces the initial dynamic stiffness of the fiber network, while improving stress redistribution within the interpenetrating film structure, consistent with the enhanced tensile strength discussed above [22]. After in situ compositing, the Enzyme-MP/PI composite film with an optimal paper-to-resin mass ratio of 1:3.5 shows a storage modulus of 1412 MPa, which remains substantially higher than that of neat PI and reflects the effective contribution of the highly crystalline fiber network to film stiffness. As the temperature approaches 120 °C, neat PI enters a viscous flow state and its storage modulus sharply decreases to below 5 MPa, reflecting a near-complete loss of film stiffness. In contrast, the Enzyme-MP/PI composite film at 1:3.5 maintains a high modulus plateau of 884 MPa even under this elevated thermal condition. This stable thermomechanical response is attributed to the densified fiber-vitrimer interpenetrating architecture, in which the highly crystalline three-dimensional fiber network maintains the stiffness of the composite film and topologically suppresses the long-range creep of the PI matrix at high temperatures.

The tan δ curves further reveal the resin-content-dependent relaxation behavior of the Enzyme-MP/PI composite films, rather than a single glass-transition process of a homogeneous polymer phase (Figure 6b). Neat PI exhibits a distinct damping peak at approximately 73.2 °C with a maximum tan δ of 0.383, corresponding to segmental relaxation of relatively unconstrained polymer chains. After incorporation of the fibrous network, the tan δ responses of the composite films become significantly broadened and strongly suppressed. This behavior indicates that polymer relaxation is governed by the coexistence of interfacially confined PI chains, less-constrained resin-rich domains, and the highly crystalline cellulose fiber network. At low PI loading, a large fraction of PI chains is located near the cellulose surface and restricted by interfacial hydrogen bonding, leading to an upward shift in the apparent tan δ maximum. With increasing PI content, the fraction of less-confined amorphous PI increases, resulting in broader relaxation behavior and partial softening at lower temperatures. For the Enzyme-MP/PI composite film with a mass ratio of 1:3.5, the suppressed tan δ peak and reduced peak intensity of 0.170 indicate restricted segmental relaxation caused by strong interfacial confinement. This typical restricted-relaxation behavior provides direct evidence for strong interfacial physicochemical interactions [22]. Specifically, the polar imine-containing chains in PI interact with hydroxyl groups on the cellulose surface to generate a high density of noncovalent anchoring sites, thereby increasing the activation barrier for segmental rearrangement of the interfacial polymer and markedly restricting local chain mobility. The slight plateau or minor increase in storage modulus above approximately 80 °C may be related to thermally activated network rearrangement within the dynamic PI phase under small-amplitude oscillatory deformation, although the overall high-temperature stiffness is mainly supported by the continuous crystalline cellulose network. At excessive PI loading, resin-rich domains become more pronounced, and the thermal softening behavior is increasingly dominated by the less-confined PI-rich phase.

Following the DMA, thermoforming experiments were used as a qualitative demonstration of the thermally activated reprocessability of the composite film above *T_g_* (Figure 6e). When the Enzyme-MP/PI composite film with a mass ratio of 1:3.5 is heated at 120 °C for 3 min, a temperature well above the matrix *T_g_* (79.7 °C), transimination within the PI network is sufficiently activated, allowing rapid dissipation of processing stress. Under external mechanical guidance, the flat composite sheet can be readily reconfigured into upright cylindrical or periodically corrugated structures. This geometric transformation originates from topological rearrangement of the vitrimer network under thermal stimulation. Meanwhile, the highly restricted microscopic relaxation and the dimensional support from the robust fibrous network effectively prevent the thermal collapse and matrix loss typically observed in neat polymers at elevated temperatures. Upon cooling to room temperature, the rearranged network is kinetically fixed, enabling the material to retain the deformed geometry without noticeable elastic recovery. Although this experiment is not intended as a quantitative shape-memory evaluation, it verifies that dynamic imine exchange enables permanent thermal reshaping of the composite film, which is distinct from the reversible softening of conventional permanently crosslinked thermosets, and serves as a fundamental basis for the subsequent interfacial self-welding as discussed in the following section.

Thermogravimetric analysis (TGA, Figure 6c,d) further defines the safe thermal processing window of the composite film. Between room temperature and 120 °C, the slight mass loss of approximately 2–5% is mainly attributed to desorption of physically adsorbed water. In the main degradation stage, pristine Enzyme-MP exhibits typical cellulose pyrolysis behavior, with a maximum degradation rate temperature (*T_max_*) of 341 °C and a char yield of 1.2%. By comparison, neat PI shows superior thermal stability, with a *T_max_* of 449 °C and a char yield of 36.9%. After compositing, the highly crosslinked PI network forms a dense thermal-physical barrier around the fibrous scaffold, as evidenced by the markedly suppressed DTG peak associated with cellulose degradation under identical testing conditions. For example, in the Enzyme-MP/PI composite film with a mass ratio of 1:3.5, the char yield at 800 °C increases to 27.5%, confirming the contribution of the PI network to thermal residue formation. More importantly, the onset temperature for 5% weight loss (*T*_5%_) remains well above 210 °C for all composite films, indicating that hot pressing at 120 °C does not induce significant thermal degradation. This thermal stability provides a reliable basis for subsequent thermally driven imine bond exchange, seamless lamination, and closed-loop recycling.

### 3.5. Thermo-Malleability and Seamless Lamination via Dynamic Imine Exchange

**Figure 7:**
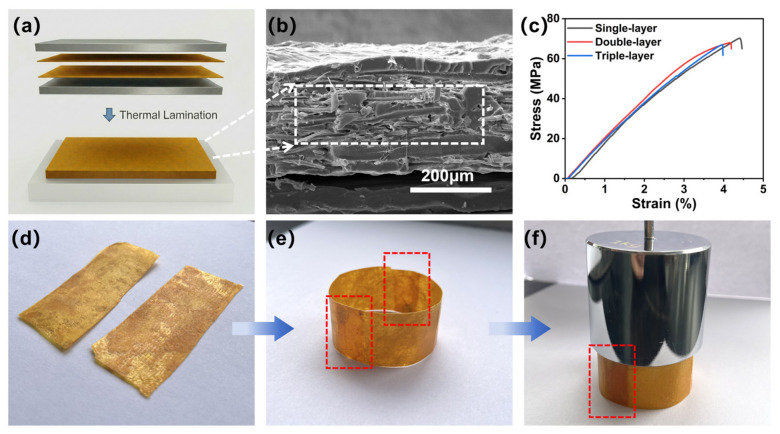
Seamless lamination and sheet-derived 3D assembly enabled by dynamic imine exchange. (**a**) Schematic illustration of thermally activated bond exchange during hot-press lamination. (**b**) Cross-sectional SEM image showing disappearance of the interface and cohesive failure behavior. (**c**) Stress–strain curves of laminated samples with increasing layer numbers. (**d**–**f**) Demonstration of thermally welded sheet assembly and geometric stability of the welded structure (The dashed box indicates the splicing junction).

Conventional fiber-based composite films and sheets are often constrained by fabrication processes and are therefore difficult to directly assemble into thick laminates or complex three-dimensional sheet-derived geometries [44]. In the present system, the incorporation of a polyimine vitrimer (PI) endows the composite film with excellent secondary processability and interfacial self-welding capability under coupled thermal–mechanical fields. As illustrated by the lamination process (Figure 7a–c), under mild hot-pressing conditions (120 °C, 5 MPa), sufficient segmental mobility enables molecular-level contact between adjacent layers. Simultaneously, dynamic imine bonds at both sides of the interface undergo efficient transimination, reconstructing a covalent topological network across the interface and thereby achieving seamless film lamination.

SEM observations of the fractured bilayer sample after tensile failure (Figure 7b) further reveal the extent of interfacial fusion. Even after tensile deformation, the original joining interface of the Enzyme-MP/PI bilayer (mass ratio = 1:3.5) is no longer distinguishable, and instead appears as a dense and homogeneous continuous phase without visible delamination or void defects. The final failure occurs via cohesive failure within the laminated film rather than adhesive failure at the interface. This microscopic evidence confirms that dynamic covalent exchange reconstructs an interfacial network that is comparable to the matrix-continuous region, thereby enabling genuine molecular-level welding.

Tensile testing (Figure 7c) quantitatively validates the robustness of this assembly strategy. As the number of layers increases from one to three, the absolute tensile force capacity increases nearly linearly, whereas the normalized tensile strength remains essentially constant at ~70 MPa, accompanied by highly consistent strain behavior. This nearly lossless thickness scaling indicates that the dynamically reconstructed interface does not introduce interlaminar defects, thereby ensuring excellent lamination stability during thickness customization.

Moreover, the thermo-malleability and interfacial welding capability of the system can be extended to the assembly of three-dimensional sheet-derived configurations. As shown in Figure 7d,e, two composite strips (80 mm × 25 mm × 1.2 mm) are locally overlapped and self-welded in situ by hot pressing to form an upright ring-shaped configuration. Under the combined thermal and pressure fields, dynamic imine bonds in the overlap region undergo topological rearrangement and generate a covalently fused interface, thereby imparting excellent joint integrity to the node. The shape-retention demonstration (Figure 7f) shows that the lightweight ring assembled from two-dimensional strips can stably support a 1 kg weight at its top without obvious buckling deformation or joint cracking. This engineering demonstration indicates that the synergy between the robust biomass fiber network and the dynamic PI matrix effectively overcomes the processing limitations of conventional thermosets, including infusibility, poor reprocessability, and difficult joining, and thus provides a reliable manufacturing strategy for customized, laminated, reprocessable, and sustainable composite sheets.

### 3.6. Anti-Wetting Behavior and Barrier Performance of Composite Films

Conventional cellulose-based materials generally exhibit poor water resistance because of the abundant hydrophilic hydroxyl groups present on both their surfaces and within the bulk. The introduction of the polyimine vitrimer (PI) network markedly reconstructs the fluid-barrier performance of the Enzyme-MP fiber network from both surface physicochemical and through-thickness barrier perspectives.

**Figure 8:**
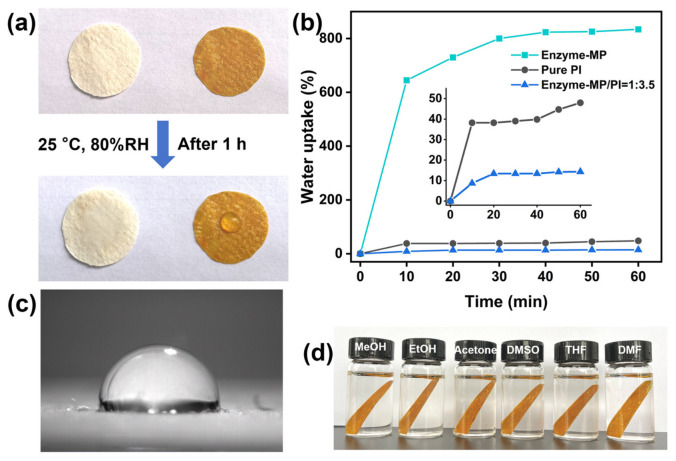
Anti-wetting behavior, water-barrier performance, and solvent resistance of Enzyme-MP/PI composite films (mass ratio = 1:3.5). (**a**) Water droplet behavior on Enzyme-MP and composite surfaces. (**b**) Dynamic water uptake comparison. (**c**) Static water contact angle measurement. (**d**) Morphological stability after immersion in various organic solvents.

The evolution of surface wettability directly reflects this transformation (Figure 8a,c). Deionized water rapidly spreads and penetrates into pristine Enzyme-MP through capillary action, exhibiting typical superhydrophilic behavior with a water contact angle close to 0°. In contrast, for the optimized Enzyme-MP/PI composite film (mass ratio = 1:3.5), the water droplet remains in a stable dome-like shape even after standing for 1 h at 25 °C and 80% RH, and the static water contact angle increases to 85°. This anti-wetting behavior, approaching the hydrophobic threshold, is mainly attributed to two factors: the intrinsically low surface free energy of the PI resin and the dense interfacial hydrogen-bonding network formed between electron-rich imine groups in PI and abundant hydroxyl groups on the fiber surface [22]. Such physicochemical anchoring effectively masks polar hydrophilic groups and thereby markedly reduces the thermodynamic affinity of the material surface toward water.

The composite film also establishes an efficient barrier against water permeation. Dynamic water uptake measurements (Figure 8b) show that Enzyme-MP exhibits a saturated water uptake of 834.0% within 1 h because of its loose and porous structure, whereas neat PI absorbs 48.0% water owing to the intrinsic free volume of the polymer network. Remarkably, the optimized composite film shows a total water uptake of only 14.3%, corresponding to a 98.3% reduction relative to pristine Enzyme-MP and even lower than that of the neat PI resin. This water-barrier effect originates from the dense fiber-vitrimer interpenetrating architecture. Specifically, the PI matrix eliminates internal pores and blocks capillary leakage pathways, while the highly crystalline and impermeable cellulose domains force water molecules to diffuse through a highly tortuous path within the polymer phase. The tight spatial complementarity between these two phases thus constructs an effective water-blocking barrier within the composite film.

Stability tests in chemically aggressive environments (Figure 8d) further demonstrate the broad solvent resistance of the optimized composite film. After immersion in a series of polar and nonpolar solvents, including methanol, ethanol, acetone, DMSO, THF, and DMF, for 72 h, the Enzyme-MP/PI composite film (1:3.5) exhibits no obvious swelling, delamination, or dissolution and retains excellent shape and dimensional integrity. This behavior is attributed to the robust multiphase network. On the one hand, the highly crystalline cellulose fiber network inherently exhibits good solvent tolerance. On the other hand, the dynamic Schiff-base network in PI remains chemically inert under ordinary solvent environments at room temperature in the absence of specific depolymerizing agents such as free amines, thereby displaying solvent resistance comparable to that of conventional thermosets.

Overall, the Enzyme-MP/PI composite film effectively overcomes the inherent water-induced disintegration of natural paper. Its excellent anti-wetting behavior, water-barrier performance, and broad solvent resistance ensure dimensional stability under aqueous and solvent-exposure conditions, providing a reliable engineering basis for replacing petroleum-based high-barrier films.

### 3.7. Dynamic Covalent Depolymerization and Closed-Loop Recycling

**Figure 9:**
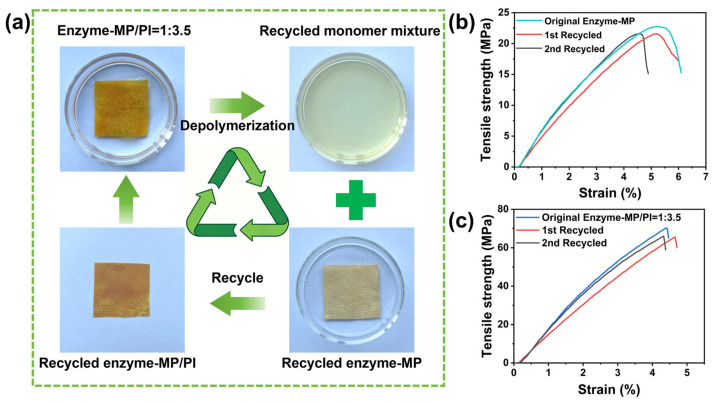
Closed-loop recycling enabled by dynamic imine chemistry. (**a**) Schematic of room-temperature depolymerization via transimination for fiber-network recovery and resin regeneration. (**b**) Stress–strain curves of recycled Enzyme-MP over multiple cycles. (**c**) Tensile performance comparison of original and regenerated composite films.

Conventional thermoset composite films are constrained by permanently crosslinked networks and therefore often face downcycling or severe environmental burdens at end of life [20]. In the present study, the dynamic Schiff-base network endows the Enzyme-MP/PI composite films with chemically enabled component-level recyclability under mild conditions. As illustrated in Figure 9a, complete depolymerization can be achieved within 1 h at room temperature by immersing the waste composite films in a DCM solution containing 1,5-pentanediamine.

The underlying mechanism is based on spontaneous transimination. Specifically, the excess free primary amine acts as a nucleophile and cleaves the crosslinked topological network of PI through amine–imine exchange, thereby converting the polymer matrix into soluble oligomeric or monomeric species and nondestructively releasing the embedded Enzyme-MP fiber network [38]. After filtration and washing, the collected degradation solution and washings are supplemented with the missing monomers according to the stoichiometric molar ratio TPTA:1,5-pentanediamine:TREN = 3:0.9:1.4, thus fully restoring the activity of the prepolymer and enabling chemical regeneration of the resin phase. The recycled fiber network and regenerated resin can then be recombined by in situ impregnation and hot pressing to fabricate regenerated composite films.

Cyclic tensile tests (Figure 9b) quantitatively confirm the nondestructive nature of this recycling strategy. After two complete depolymerization–reprocessing cycles, the tensile strength of the recycled mulberry paper remains stable at approximately 21 MPa, showing no significant deterioration relative to the original fiber network (22.7 MPa). This excellent retention of tensile strength confirms the high chemical selectivity of the room-temperature transimination process, which selectively deconstructs the synthetic polymer network while preserving the molecular backbone and crystalline structure of natural cellulose. In addition, the regenerated composite films fabricated from the recycled fiber network and regenerated resin maintain key tensile properties highly comparable to those of the virgin material, including a tensile strength of approximately 68 MPa versus 70.3 MPa for the original composite film. This stable tensile performance indicates that the regenerated PI precursor still possesses sufficient permeability toward the porous fiber network and is capable of re-establishing a dense interfacial hydrogen-bonding network with the cellulose surface during secondary hot pressing and curing.

Although the present recycling protocol verifies the chemical feasibility of full-component recovery through dynamic imine exchange, dichloromethane and excess diamine were used here as model depolymerization agents. Their environmental and health impacts should be considered in future process optimization. Accordingly, the sustainability-related claims in this work are limited to the demonstrated closed-loop recyclability of the material components, while greener solvent systems, bio-derived amine feedstocks, solvent recovery, and life-cycle assessment will be pursued in future work.

Overall, this room-temperature depolymerization strategy based on dynamic covalent chemistry demonstrates proof-of-concept closed-loop recovery of Enzyme-MP/PI composite films. By avoiding extreme thermal treatment and harsh acidic or alkaline conditions, the process realizes nondestructive and bidirectional closed-loop recovery of both the cellulose fiber network and the matrix phase, thereby providing a chemical basis for future cradle-to-cradle material circulation in recyclable plastic-replacement films.

## 4. Conclusions

This work reports an enzyme-treated mulberry paper/polyimine (Enzyme-MP/PI) interpenetrating composite film that integrates high tensile performance, thermomechanical robustness, thermo-malleability, and chemically enabled full-component recovery within a single material system. By combining a highly crystalline, interwoven cellulose fiber network with a dynamic polyimine vitrimer matrix, a dense fiber-vitrimer interpenetrating architecture is established through capillary densification and strong interfacial hydrogen bonding. The resulting interfacial confinement effect significantly enhances stress transfer efficiency, leading to a 195% increase in tensile strength relative to neat PI (up to 70.3 MPa), while maintaining sufficient thermomechanical stability for hot-pressing, lamination, and dimensional retention at 120 °C. Moreover, dynamic imine exchange within the vitrimer network enables thermo-malleability, allowing seamless lamination, thickness-scalable assembly, and the fabrication of complex three-dimensional sheet-derived configurations at 120 °C without interfacial degradation. Simultaneously, the interpenetrating architecture forms an effective multiscale barrier, markedly suppressing water uptake to 14.3% and providing robust resistance to fluid penetration. Importantly, the dynamic covalent network enables selective room-temperature transimination, allowing component-level separation of the highly crystalline cellulose fiber network and chemical regeneration of the polymer matrix at the monomer level. The regenerated composite films retain stable tensile performance over multiple recycling cycles. Overall, this study establishes a dynamic interfacial engineering strategy that integrates high-strength film performance with end-of-life recyclability, offering a potential pathway toward cradle-to-cradle material circulation for next-generation plastic-replacement composite films.

## Data Availability

The original contributions presented in this study are included in the article. Further inquiries can be directed to the corresponding author.
